# Robot‑assisted surgical management of mid‑urethral sling erosion into the bladder using transperitoneal robotic extensive approach for total mesh excision

**DOI:** 10.20452/wiitm.2024.17939

**Published:** 2025-03-21

**Authors:** Rafał B. Drobot, Marcin Lipa, Jędrzej J. Skorupka, Artur A. Antoniewicz

**Affiliations:** Urology Department, Institute of Medical Sciences, Faculty of Medicine, Collegium Medicum, Cardinal Stefan Wyszyński University in Warsaw, Warszawa, Poland; Department of Urology and Urological Oncology, Multidisciplinary Hospital in Warsaw‑Miedzylesie, Warszawa, Poland

**Keywords:** mid‑urethral sling erosion, robot‑assisted surgery, tension‑free vaginal tape excision, transobturator tape excision, urine incontinence surgery complications

## Abstract

Robot‑assisted surgical management of mid‑urethral sling (MUS) erosion into the bladder represents a novel approach in urological surgery. This study reports the first 2 cases in Poland treated using the transperitoneal robotic extensive approach for total mesh excision (TREATME). Both procedures were performed successfully, with complete mesh removal. No intra‑ or postoperative complications occurred, and conversion to open surgery was not required. These initial findings suggest that TREATME may be a feasible and safe option for managing complex MUS complications, necessitating further evaluation in larger studies.

## INTRODUCTION

Mid‑urethral slings (MUSs), including transobturator tape (TOT) and tension‑free vaginal tape (TVT), are widely recognized as effective treatments for stress urinary incontinence (SUI). Long‑term objective success rates range from 53.6% for TVT to 60.1% for TOT, with subjective success rates reaching up to 75.6% for TVT over a median follow‑up of 48 months (range, 24–60 months).^1^ These minimally invasive techniques have significantly improved SUI management, enhancing treatment outcomes and patient quality of life. Notably, TOT has been reported to reduce International Consultation on Incontinence Questionnaire–Urinary Incontinence Short Form scores by 8.78 points over 5 years, with TVT achieving comparable out‑ comes. Consequently, over 85% of patients report improvements in social confidence, psycho‑ logical well‑being, and sexual satisfaction.^2^^,^^3^ Despite their success, MUS procedures carry a risk of rare but serious complications, such as mesh erosion, bladder injury, chronic pain syndrome, or recurrent infections, which may require com‑ plex surgical management. Managing intravesical sling erosion is particularly challenging owing to the delicate surrounding structures and the risk of incomplete removal, potentially leading to symptom recurrence. Conventional open or laparoscopic approaches often involve significant morbidity and technical limitations in such cases. Robot‑assisted surgery (RAS), with its advanced range of motion, tremor filtration, and superior magnification, enables precise dissection in confined spaces, making it a promising alter‑ native for these complex procedures.

## AIM

This study presents a novel RAS technique for managing MUS erosion into the bladder— transperitoneal robotic extensive approach for total mesh excision (TREATME). It evaluates the feasibility and safety of this approach based on 2 retrospective initial cases, marking the first documented implementation of this technique in Poland, with a comparative narrative analysis of outcomes against alternative surgical methods reported in the literature.

## MATERIALS AND METHODS

Patients and study de‑ sign Between June 2023 and July 2024, 2 women with cystoscopically confirmed intravesical MUS erosion and secondary bladder stones underwent robot‑assisted total mesh excision.

**FIGURE 1:**
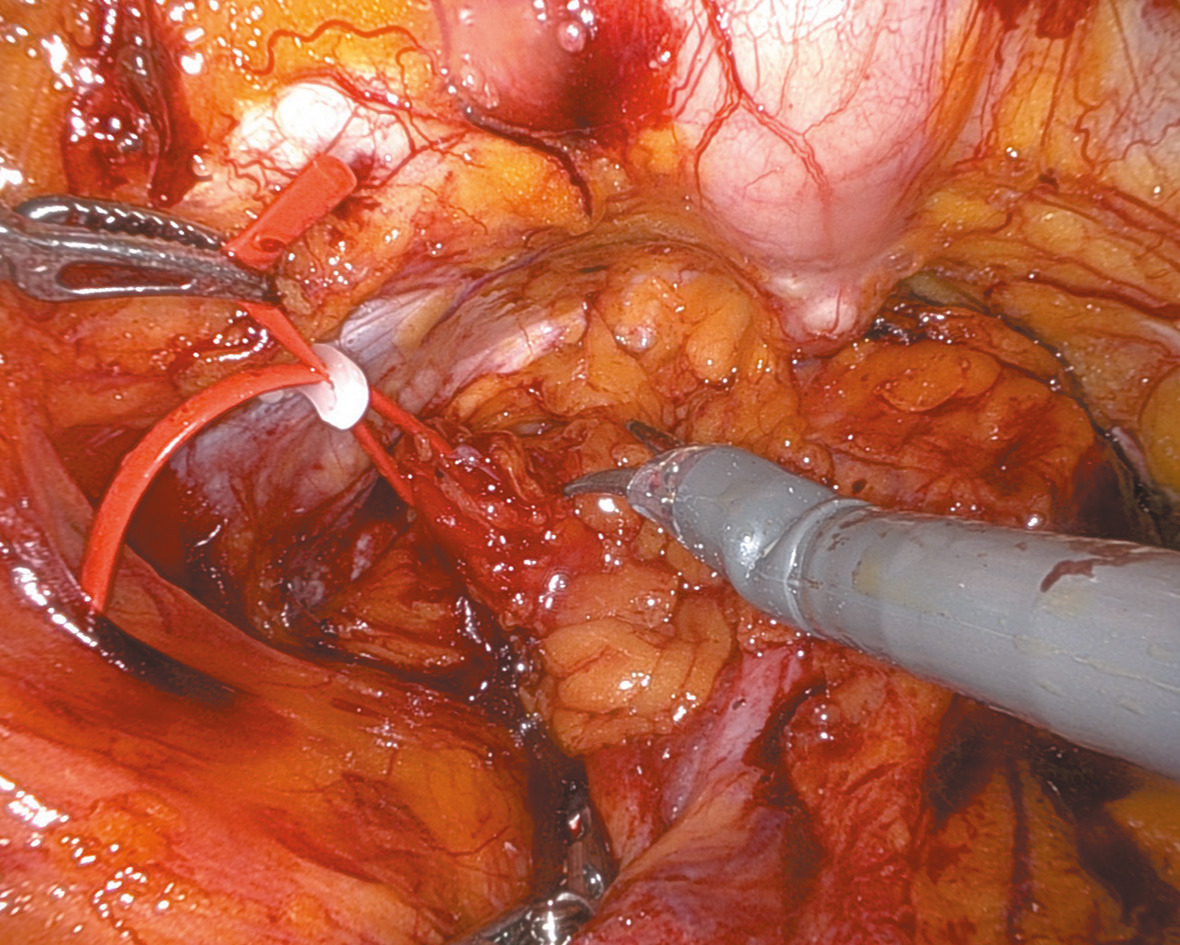
Left transobturator tape arm

**FIGURE 2:**
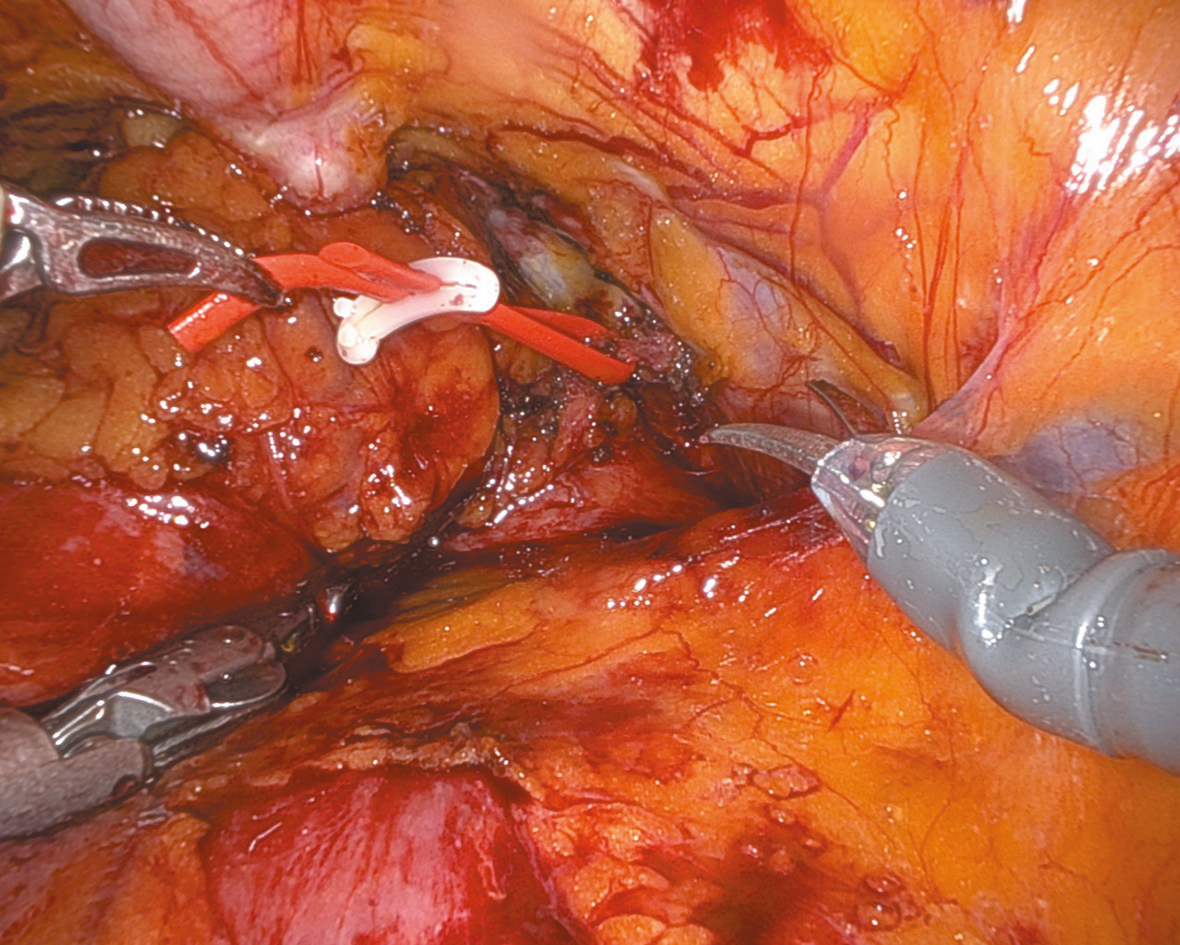
Right transobturator tape arm

The slings, placed over 12 months prior for SUI, resulted in chronic pelvic pain and irritative lower urinary tract symptoms in one patient, and recur‑ rent urinary tract infections in the other. The robotic approach was selected based on institutional expertise and encouraging outcomes reported in the literature. All procedures were performed by 2 certified console surgeons (AAA, RD) using the da Vinci X system (Intuitive, Sunnyvale, California, United States).

Informed consent was obtained from both patients for the surgery and subsequent publication of their case details. Feasibility was assessed based on surgical duration (min), intraoperative blood loss (ml), and conversion rate to open surgery. Safety evaluation included the analysis of intra‑ and postoperative complications classified using the Clavien–Dindo system, length of hospital stay (days), and 30‑day readmission rates. Comparative narrative analysis focused on sur‑ gical outcomes, including complete sling removal without residual erosion, complication rates stratified by Clavien–Dindo grades, and long‑term results, such as symptom recurrence and functional recovery. This retrospective study was registered in the research registry (ID: 10970); detailed in‑ formation are available at https://researchregis‑ try.knack.com/research‑registry#home/registrat iondetails/67843bd138d97e02c0fcbc1f/.

### Preoperative cystoscopy with cystolithotrypsy

Pre‑ operative urethrocystoscopy was performed to identify the ureteral orifices and assess the position of the eroded mesh relative to the orifices. If the erosion site was located near a ureteral orifice, a double‑J stent was placed intraoperatively to protect the orifice during the second surgical stage. The calculus formed on the mesh, which served as a nidus for crystallization, was treated using mechanical lithotripsy with a nephroscope and the Swiss LithoClast system (Electro Medical Systems, Nyon, Switzerland). This method is pre‑ ferred over laser lithotripsy to prevent thermal damage to the mesh, as such damage could com‑ plicate subsequent robotic removal of MUS. Fol‑ lowing fragmentation, cystolitholapaxy was per‑ formed to remove bladder debris. Intraoperative transvaginal ultrasound was utilized to precise‑ ly delineate mesh localization and guide its me‑ ticulous excision.

### Transperitoneal robotic extensive approach for total mesh excision

**FIGURE 3:**
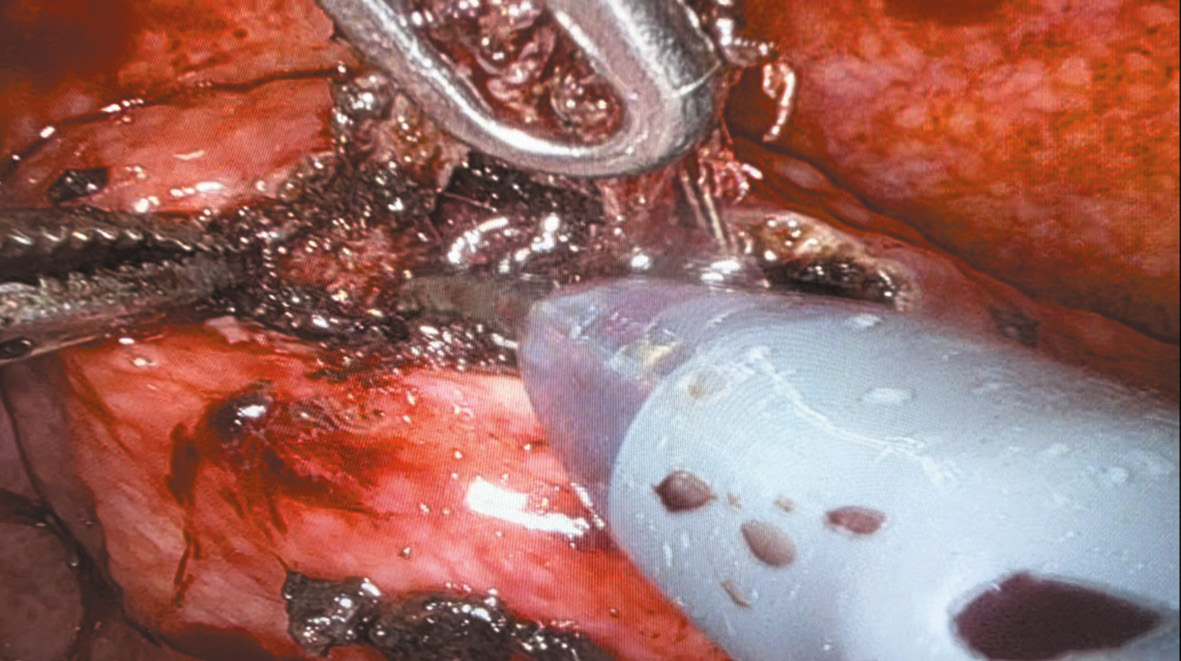
Meticulous excision of eroded mesh

**FIGURE 4:**
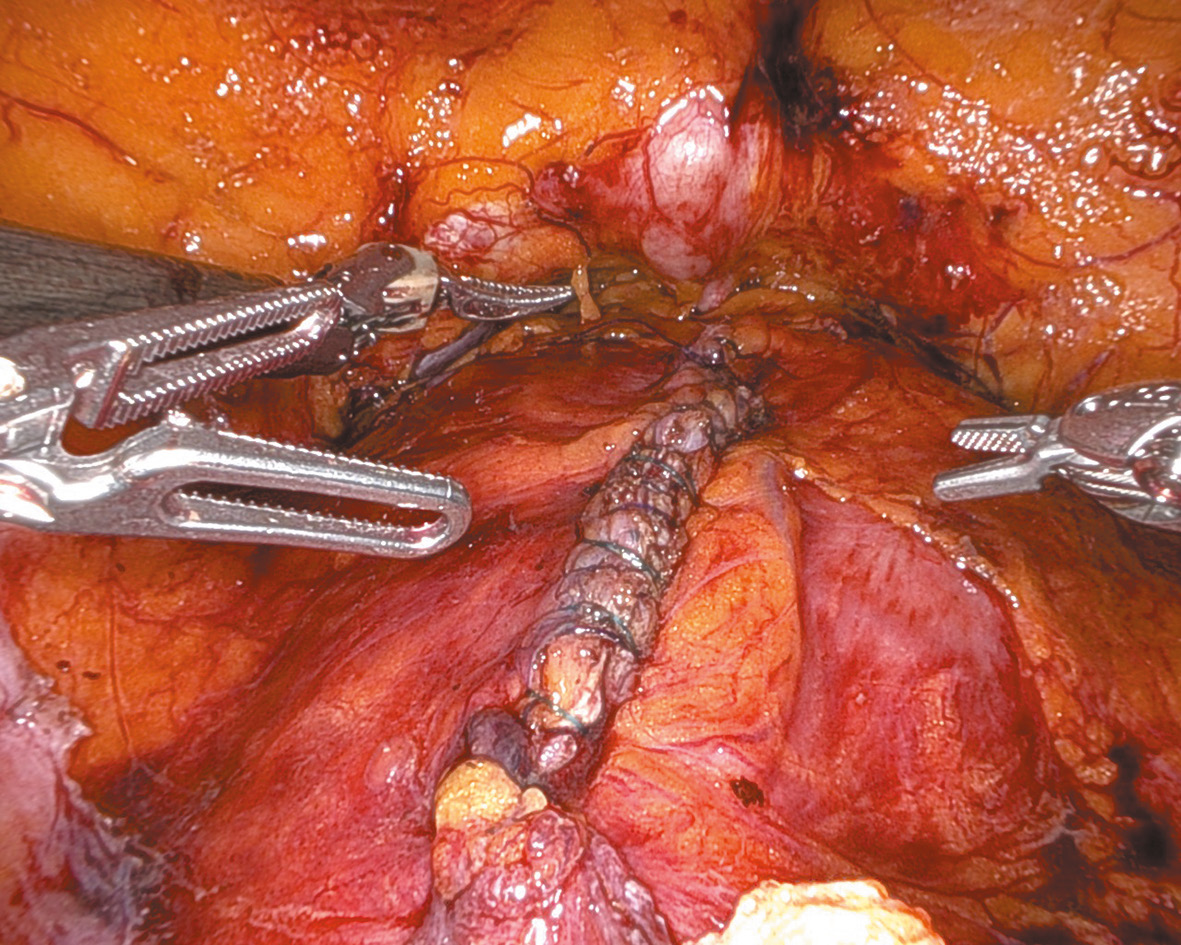
Double‑layer cystorrhaphy with a leak test

Under general anesthesia, the patient was positioned supine in a −30 ° Trendelenburg position. Four 8‑mm robotic tro‑ cars were placed transversely above the umbilicus, and one 11‑mm laparoscopic trocar was inserted laterally above the iliac crest for the assistant. The parietal peritoneum was incised transverse‑ ly over the bladder through the median and lateral umbilical ligaments, which were coagulated, to access the preperitoneal space below the pubic symphysis to the pelvic fascia. In the TVT case, the tape arms were visualized vertically beneath the bladder neck. Conversely, for TOT, the arms were identified transversely, entering the bladder neck area laterally from the obturator fora‑ men (FIGURE 1 and FIGURE 2). Subsequently, the extravesical tape segments between the abdominal or pelvic wall and the bladder neck were dissected. After filling the bladder with saline via a Foley catheter, a midline cystotomy was performed, and the bladder walls were suspended to enhance exposure. The intravesical erosion site of the tape and the ureteral orifice locations were identified (Video 1). The bladder mucosa was incised later‑ ally toward the tape arms, which were dissected free from the space between the anterior vaginal wall and the posterior bladder wall FIGURE 3. Extravesical tape portions anchored to the bladder wall or pelvic structures were subsequently ex‑ cised. The tape was sectioned, and the fragments were evacuated by the assistant. The bladder mu‑ cosa was closed with continuous sutures, ensuring that the knot was securely buried within the tis‑ sue. Two‑layer cystorrhaphy was performed, and bladder integrity was confirmed by a saline leak test FIGURE 4. A drain was placed in the Retzius space, and the Foley catheter remained in place for 10 to 14 days to support low‑pressure blad‑ der healing.

## RESULTS

### Feasibility 

The TREATME approach may be a viable option for managing complex MUS complications, as demonstrated by its application in 2 challenging cases. The first case involved a 60‑year‑old woman who underwent TVT explantation on June 27, 2023. The procedure lasted 125 min, with 90 min spent on the robotic console. The second patient, a 63‑year‑old woman who underwent TOT explantation on July 3, 2024, had a shorter operative duration of 65 min, including 50 min of console time. The estimated blood loss was 250 ml for patient 1 and 100 ml for patient 2. The first patient, whose urine culture was sterile preoperatively, received a single dose of third‑generation cephalosporin during the perioperative period. In contrast, the second patient had a preoperative culture indicating an extended‑spectrum β‑lactamase–producing bacterial strain. She was treated with a carbapenem, initiated 48 hours before surgery and continued throughout the perioperative period. Neither patient developed postoperative symptoms of infection. The preoperative microbiological and perioperative laboratory findings and targeted antibiotic regimens are summarized in TABLE 1.

### Safety

The safety profile of the TREATME ap‑ proach appears favorable for the management of MUS erosions with associated bladder stones. Both patients had uneventful perioperative courses with no complications (Clavien−Dindo grade 0). The first patient was hospitalized for 7 days, while the second one was hospitalized for 9 days. Drains placed in the Retzius space were removed on the second postoperative day. Both patients were discharged with indwelling Fol‑ ey catheters, which were subsequently removed on postoperative day 10 (patient 1) and 12 (pa‑ tient 2). Tailored antibiotic regimens effective‑ ly reduced the risk of complications. Details of the postoperative management of MUS erosions are summarized in TABLE 2.

## DISCUSSION 

### Complete excision and precision

RAS enables precise excision of eroded MUS, addressing both intravesical and extravesical components. Fong et al^4^ reported an 83.3% rate of near‑complete mesh removal among 30 patients, with a me‑ dian console time of 148 min. Olive et al^5^ de‑ scribed successful removal of a 6‑cm mesh segment along with bladder stones, achieved without postoperative complications or residual mesh fragments. These results highlight the ability of the robotic system to navigate complex anatomical regions, including the trigone and ureteral orifices, which present challenges for endoscop‑ ic and laparoscopic techniques.^6^^,^^7^

### Operative metrics and efficiency

Data on operative time and blood loss indicate consistent performance of the robot‑assisted technique in managing MUS erosion. Macedo et al^6^ documented an average operative time of 138 min with 100 ml of blood loss. In comparison, Fong et al^4^ reported a median operative time of 240 min, likely re‑ flecting cases with extensive extravesical dissection. Blood loss across studies ranged from 100 to 140 ml.^4^^,^^6^^,^^8^ The blood loss ratio of 2.5:1 in the first case (TVT explantation) vs the second case (TOT explantation) is likely attributable to anatomical differences (TVT slings in the vascular‑rich retro‑ pubic space vs TOT in the less vascular obturator foramen) and the learning curve of TREATME. Material‑related factors can be excluded, as both slings are made of polypropylene. Laparoscopic approaches for MUS removal, as reported by Siddharth et al,^9^ showed operative times ranging from 165 to 180 min, with notable variability observed in more complex cases. By contrast, robot‑assisted techniques exhibited a learning curve that stabilized after 15–20 cases, reducing operative times to less than 150 min when the procedure was performed by experienced surgeons.^10^ An operative time ratio of 1.9:1 in the first institutional cases supports this observation, indicating improved efficiency with experience. No significant challenges were encountered, confirm‑ ing feasibility with adequate training.

**TABLE 1:** Pre- and perioperative parameters

Parameter		TVT case	TOT case
Hemoglobin, mmol/l	Preoperative	7.5	Preoperative: 7.2
	Postoperative	6.5	Postoperative: 7.8
Creatinine, mg/dl	Preoperative	0.85	Preoperative: 0.92
	Postoperative	0.89	Postoperative: 0.98
Preoperative urine culture		Sterile	*Escherichia coli* ESBL (+), 10⁶ CFU/ml
Antibiotic therapy		Ceftriaxone (2 g, single dose)	Meropenem (1 g every 8 h for 48 h preoperatively, intraoperatively, and postoperatively)

SI conversion factors: to convert hemoglobin to g/l, multiply by 16.11; creatinine

to μmol/l, by 88.4.

Abbreviations: CFU, colony‑forming units; ESBL, extended‑spectrum β‑lactamase;

TVT, tension‑free vaginal tape; TOT, transobturator tape

**TABLE 2:** Postoperative outcomes

Parameter	TVT case	TOT case
Drain removal, POD	2	2
Catheter removal, POD	10	12

Abbreviations: POD, postoperative day; others, see TABLE 1

### Functional and symptomatic outcomes

Robotic approaches result in favorable functional outcomes for patients with MUS erosion. Chan et al^8^ reported a significant improvement in lower urinary tract symptoms in 84.4% of patients, with 9.4% requiring additional interventions for SUI.^8^ Olive et al^5^ reported a 93% resolution rate for overactive bladder symptoms, and no new cases of stress incontinence postoperatively. Conversely, Souders et al^11^ found that transvaginal mesh excision reduced pain in 50% of cases, while 21% of patients reported persistent symptoms. In our cohort, no complications or adverse events were observed during follow‑up periods of 20 months for the first patient (TVT explantation, June 2023) and 8 months for the second (TOT explantation, July 2024). No additional medications were re‑ quired. Bladder neck competency was not formally assessed, but continence remained stable, as indicated by unchanged pad usage. However, longer‑term data beyond these intervals remain limited, precluding a full assessment of outcome durability, including risks of symptom recurrence, late complications, or the need for additional in‑ terventions. Extended monitoring continues, with results to be reported subsequently. These findings suggest that achieving complete mesh excision through advanced robotic techniques plays a crucial role in symptom improvement.

### Complication rates and risk mitigation

Complication rates reported for robotic approaches are relatively low across studies. Chan et al^8^ observed urinary leaks in 6.2% of cases, with no reported cases of vesicovaginal fistulas (Grade IIIb acording to the Clavien−Dindo classification). Preoperative ureteral stenting, as described by Popat et al,^12^ was employed to protect adjacent structures, particularly in the cases involving erosion near the trigone. However, endoscopic methods were associated with a higher recurrence rate of 21% and a 9% incidence of vesicovaginal fistulas.^13^ Preoperative planning with translabial ultrasound or 3‑dimensional imaging, as noted by Przudzik et al,^14^ aids in identifying the ex‑ tent and location of mesh erosion, potentially reducing the risk of complications during surgical interventions.

### Comparison with laparoscopic and endoscopic techniques

Outcomes of robotic approaches have been compared with those of laparoscopic and endoscopic methods regarding operative com‑ pleteness. Zamecnik et al^7^ found that laparoscopic techniques are effective in isolated intra‑ vesical erosions but may leave residual mesh in 30% of cases involving extravesical components. Karim et al^13^ reported that the endoscopic laser‑based approach was faster, with operative times ranging from 90 to 120 minutes, but often left residual fragments (up to 25% of cases). By contrast, only 16.7% of cases using the robotic approach involved partial or incomplete mesh removal, demonstrating its capability to manage complex anatomical scenarios with a high level of precision.^4^

### Cost implications and resource allocation 

The financial aspects of the robotic approach are an important consideration. Macedo et al^6^ estimated the additional cost of robotic procedures at USD 800–1000, as compared with laparoscopic methods. However, Olive et al^5^ reported a reduction in the length of hospital stays, with robotic cases averaging 2.8 days, as compared with 4.3 days for laparoscopic approaches. Gurol‑Urganci et al^15^ emphasized that incomplete excision often necessitates secondary interventions, contributing to higher cumulative costs over time. These findings suggest that the higher initial cost of TREATME (equipment, systems, training) may be offset by reduced hospital stays, faster recovery, and fewer reinterventions, potentially lowering expenses. Cost‑effectiveness of this approach, as compared with laparoscopy, requires analysis of direct (personnel, operative time) and indirect costs (complications), and an economic evaluation to quantify savings and cost‑benefit ratio for patients and health care systems. In Poland, TREATME is confined to specialized centers, with reimbursement by the National Healthcare System unavailable and non standardized, restricting access for publicly funded patients and impacting societal cost‑effectiveness. Future health economic analyses should guide reimbursement and implementation policies.

### Patient selection and surgical planning

Accurate patient selection is critical for optimizing outcomes. Przudzik et al^14^ highlighted the use of translabial ultrasound as an effective tool for identifying the extent and location of mesh erosion, particularly in complex cases, aiding in the selection of the most appropriate surgical approach. Similarly, Macedo et al^6^ emphasized the importance of evaluating extravesical involvement to inform the choice of technique. While simpler methods may suffice for isolated intravesical erosion, robotic techniques have been recommended for ex‑ tensive or complex erosions owing to their superior precision and adaptability.^12^

Long‑term outcomes and recurrence rates

The durability of outcomes following a robotic approach requires further investigation. Souders et al^11^ reported a 21% recurrence rate of pain following transvaginal excision, as compared with significantly lower rates with robotic approaches. Chan et al^8^ reported a 12% recurrence of SUI after RAS, suggesting the need for follow‑up care in some patients. Trump et al^16^ demonstrated com‑ plete resolution of pelvic pain and voiding dysfunction in a patient after robotic retropubic MUS removal, with the patient achieving full function‑ al recovery and discontinuation of all pain medi‑ cations during the postoperative period.

### Areas for future research 

Although RAS offers promising outcomes, limitations remain regarding its accessibility and training requirements. Future research should focus on improving accessibility through cost‑sharing models and technological advancements. Additionally, randomized trials comparing the robotic approach with emerging hybrid techniques and long‑term quality‑of‑life studies are essential to further define the po‑ tential benefits and limitations of these surgical methods.^13^^,^^15^ The integration of advanced imaging modalities, such as pelvic ultrasound and 3‑dimensional imaging, into preoperative planning represents a significant area for innovation. Lou et al^17^ demonstrated that these tools enhance surgical precision and support patient‑centered care by enabling individualized treatment strategies.^17^ Hybrid approaches combining robotic precision for complex cases with less invasive or conservative methods for simpler ones could further optimize outcomes while balancing risks and costs. Conservative management of mesh erosion, as

highlighted by O’Kane et al,^18^ is another area requiring further investigation. While tradition‑ ally underutilized, nonsurgical approaches may provide a viable alternative for selected patients who decline invasive interventions or present with contraindications. Research should evaluate the long‑term efficacy and safety of such strategies compared with surgical removal. Additionally, Güler et al^19^ emphasized the importance of early detection and standardized imaging protocols to minimize complications and improve clinical outcomes. Developing evidence‑based guidelines for the use of advanced imaging in routine preoperative assessments could enhance the success rates of both robotic and hybrid approaches. Lastly, Doyle et al^20^ highlighted the nuanced outcomes of partial vs total mesh removal, with partial excisions associated with lower rates of postoperative SUI. Future studies should explore the criteria for selecting partial or total excision based on patient profiles, erosion severity, and functional outcomes. By addressing these areas, future research could further refine the management of MUS erosion, ensuring that interventions remain both effective and accessible while minimizing patient burden.

## CONCLUSIONS 

TREATME is a novel and effective surgical approach for managing complex MUS complications, particularly intravesical erosion. This technique enables precise and complete mesh removal, addressing both intra‑ and extravesical components while minimizing the risk of recur‑ rence or complications. The feasibility and safety outcomes from the initial cases, compared with current surgical excision techniques, suggest that TREATME has the potential to become a standard treatment option for challenging cases. Given that this study only reports 2 cases, the promising initial results regarding the feasibility and safety of this robotic technique require confirmation through further research involving a larger patient cohort, adhering to the Strengthening the Reporting of Observational Studies in Epidemiology or Consolidated Standards of Reporting Trials guidelines to definitively establish its efficacy, reliability, and long‑term benefits in improving patient outcomes and surgical efficiency.

## VIDEO

The video file is available online at https://dx.doi.org/10.20452/ wiitm.2025.17939.

## Custom Section

Drobot RB, Lipa M, Skorupka JJ, Antoniewicz AA. Robot‑assisted surgical management of mid‑urethral sling erosion into the bladder using transperitoneal robotic extensive approach for total mesh excision. Wideochir Inne Tech Maloinwazyjne. 2025; 20: 118-123. doi:10.20452/wiitm.2024.17939
